# LncRNA-Disease Association Prediction Using Two-Side Sparse Self-Representation

**DOI:** 10.3389/fgene.2019.00476

**Published:** 2019-05-28

**Authors:** Le Ou-Yang, Jiang Huang, Xiao-Fei Zhang, Yan-Ran Li, Yiwen Sun, Shan He, Zexuan Zhu

**Affiliations:** ^1^Guangdong Key Laboratory of Intelligent Information Processing and Shenzhen Key Laboratory of Media Security, Shenzhen University, Shenzhen, China; ^2^FJKLMAA (Fujian Key Laborotary of Mathematical Analysis and Applications), Fujian Normal University, Fuzhou, China; ^3^College of Computer Science and Software Engineering, Shenzhen University, Shenzhen, China; ^4^School of Mathematics and Statistics and Hubei Key Laboratory of Mathematical Sciences, Central China Normal University, Wuhan, China; ^5^School of Medicine, Shenzhen University, Shenzhen, China; ^6^School of Computer Science, University of Birmingham, Birmingham, United Kingdom

**Keywords:** lncRNAs-disease associations prediction, computational approaches, sparse representation, lncRNA similarity, disease similarity

## Abstract

Evidences increasingly indicate the involvement of long non-coding RNAs (lncRNAs) in various biological processes. As the mutations and abnormalities of lncRNAs are closely related to the progression of complex diseases, the identification of lncRNA-disease associations has become an important step toward the understanding and treatment of diseases. Since only a limited number of lncRNA-disease associations have been validated, an increasing number of computational approaches have been developed for predicting potential lncRNA-disease associations. However, how to predict potential associations precisely through computational approaches remains challenging. In this study, we propose a novel two-side sparse self-representation (TSSR) algorithm for lncRNA-disease association prediction. By learning the self-representations of lncRNAs and diseases from known lncRNA-disease associations adaptively, and leveraging the information provided by known lncRNA-disease associations and the intra-associations among lncRNAs and diseases derived from other existing databases, our model could effectively utilize the estimated representations of lncRNAs and diseases to predict potential lncRNA-disease associations. The experiment results on three real data sets demonstrate that our TSSR outperforms other competing methods significantly. Moreover, to further evaluate the effectiveness of TSSR in predicting potential lncRNAs-disease associations, case studies of Melanoma, Glioblastoma, and Glioma are carried out in this paper. The results demonstrate that TSSR can effectively identify some candidate lncRNAs associated with these three diseases.

## 1. Introduction

Long non-coding RNAs (lncRNAs), which are a class of non-coding transcripts with the lengths longer than 200 nucleotides (Derrien et al., 2012; Harrow et al., 2012; Guttman et al., 2013; Chen et al., 2016b), have been proven to be involved in various biological processes (Chen et al., 2012, 2016b, 2018) and closely correlated with the development of complex diseases, such as cancers and rheumatic diseases (Bussemakers et al., 1999; Managadze et al., 2011; Bhartiya et al., 2012; Schonrock et al., 2012; Li et al., 2013; Lu et al., 2013; Zhao et al., 2014; Chen et al., 2016b). For example, studies have revealed the roles of lncRNAs in regulating gene expression (Taft et al., 2010; Wapinski and Chang, 2011). As the development of complex diseases are closely related to the mutations and abnormalities of lncRNAs, to understand the pathogenesis of human diseases systematically, and identify the biomarkers of disease progression and prognosis, it is important to predict the potential associations between diseases and lncRNAs (Chen et al., 2016b; Yu et al., 2018). However, only a small number of lncRNA-disease associations have been validated. Therefore, efficient methods for predicting the associations between lncRNAs and diseases are emergent needed (Lu et al., 2018).

In recent years, identifying the associations between diseases and lncRNAs has attracted a lot of attentions (Chen and Yan, 2013; Lu et al., 2018). Prediction methods based on biological experiments or computational approaches are proposed to undertake this task. Due to the limitations of biological experiments such as time-consuming and expensive in cost, computational approaches provide an alternative for biological experiments and have been widely used to identify the associations between lncRNAs and diseases (Chen et al., 2016b). Existing computational approaches for association prediction can be roughly classified into three categories. The first category is based on machine learning approaches. These models predict the associations between diseases and lncRNAs based on known lncRNA-disease associations. For example, Chen et al. proposed a semi-supervised learning-based method named Laplacian Regularized Least Squares for LncRNA-disease Association (LRLSLDA) (Chen and Yan, 2013) to predict the associations between diseases and lncRNAs. Zheng et al. formulated the problem of association prediction as a matrix factorization problem and introduced a collaborative matrix factorization model (CMF) (Zheng et al., 2013) to predict the associations. However, the performance of machine learning-based methods depend on the choice of hyperparameters such as the dimensionality of the latent space in matrix factorization-based methods, and the suitable values for these hyperparameters are usually previously unknown and hard to determine.

The second category is based on random walk. These models identify potential lncRNA-disease associations by integrating known associations between diseases and lncRNAs and similarities among diseases and lncRNAs. For example, Zhou et al. predicted the associations between diseases and lncRNAs by implementing random walk with restart on the constructed similarity networks among lncRNAs and diseases (Zhou M. et al., 2015). The third category is based on data integration. These models focus on integrating multiple heterogeneous data sources. For example, Lu et al. (2018) developed a model named SIMCLDA for identifying the associations between diseases and lncRNAs based on disease-gene and gene-gene ontology associations. However, the above methods rely heavily on the similarity networks or external information (e.g., similarity networks among diseases and lncRNAs, and gene-gene associations) that are inferred based on predefined metrics. Moreover, the information extracted from other databases or data platforms may include some irrelevant or noise information that may mislead the prediction of associations.

To address the above problems, in this paper, we introduce a novel two-side sparse self-representation (TSSR) model for lncRNA-disease association prediction. Based on known lncRNA-disease associations, our model can adaptively learn two non-negative sparse self-representation matrices which capture the intra-similarities among lncRNAs and diseases respectively. Moreover, our model could also drawn support from the intra-associations among disease and lncRNAs that derived from external information of lncRNAs and diseases to generate more accurate estimation of the representation matrices. Experiment results on three real datasets demonstrate that compared with six state-of-the-art association prediction algorithms, our TSSR model could achieve more accurate prediction results. Furthermore, case studies on three cancers (i.e., Glioblastoma, Glioma, and Melanoma) also demonstrate the effectiveness of TSSR in predicting the associations between lncRNAs and diseases. The source code of TSSR is available at https://github.com/Oyl-CityU/TSSR.

The rest of this paper is organized as follows. In section 2, we formulate our two-side sparse self-representation model and introduce a relaxed Majorization-Minimization algorithm to solve the optimization problem. The experiment results and case studies are given in section 3. In section 4, we conclude our works.

## 2. Methods

### 2.1. Notations and Problem Statement

In this paper, we use $D={\left\{d_{i}\right\}}_{i=1}^{m}$ to represent the set of lncRNAs and $T={\left\{t_{j}\right\}}_{j=1}^{n}$ to represent the set of diseases, where *m* and *n* denote the number of lncRNAs and the number of diseases, respectively. A binary matrix $Y=\left[Y_{ij}\right]\in {\left\{0,1\right\}}^{m\times n}$ is introduced to represent the associations between lncRNAs and diseases, where *Y*_*ij*_ = 1 if there is an association between lncRNA *d*_*i*_ and disease *t*_*j*_, and *Y*_*ij*_ = 0 otherwise. Note that there are two reasons that may lead to *Y*_*ij*_ = 0. The first reason is that it has been experimentally verified that there is no association between *d*_*i*_ and *t*_*j*_. The second reason is that whether there is an association between *d*_*i*_ and *t*_*j*_ is still unknown. Therefore, we usually refer to the zero elements in *Y* as unknown pairs. The lncRNA-disease association prediction problem can be formulated as the problem of predicting the scores of unknown pairs in *Y*, which can be used for ranking the pairs. In this study, we first rank the unknown pairs in *Y* based on the predicted scores in descending order, and then select the top-ranked pairs as potential association pairs.

In particular, unlike matrix factorization methods that project lncRNAs and diseases into a shared latent space and predict lncRNA-disease associations based on the inner product of their latent vectors, we try to learn the intra-similarities among lncRNAs and diseases from the observed associations in *Y*, and utilize the learned similarity matrices to reconstruct *Y* and thus predict the scores of unknown pairs in *Y*. Here, instead of using predefined metrics to construct the similarity matrices of lncRNAs and diseases (which makes the predicted results sensitive to the selected metrics and input data), we introduce a novel two-side sparse self-representation (TSSR) model to adaptively learn the intra-similarities among lncRNAs and diseases from the observed associations in *Y*, and effectively utilize external information of lncRNAs and diseases to enhance the prediction performance.

### 2.2. Two-Side Sparse Self-Representation Model

Sparse representation techniques which focus on finding a sparse representation of a sample in the form of a linear combination of basic elements (also called atoms) in a dictionary, have been widely used to numerous applications such as computer vision and machine learning (Zhang et al., 2015). In traditional sparse representation models, the objective is to solve the following problem

(1) $$\begin{array}{r} \underset{\text{x}}{\min}||\text{x}|{|}_{0}\;s.t.\;\text{y}=D\text{x}. \end{array}$$

where ||·||_0_ denotes *L*_0_ norm, **y** ∈ *R*^*m*×1^ is a sample vector, *D* is a *m* × *l* matrix which denotes the dictionary and **x** ∈ *R*^*l*×1^ is the sparse representation coefficient of **y**. In practice, *L*_0_ norm is usually replaced with *L*_1_ norm to make the above problem (1) solvable in polynomial time. Since the above problem (1) needs to take extra time to construct the dictionary *D* and has not data-adaptiveness. Many approaches are proposed to employ the dataset itself as the dictionary, which results in the following sparse self-representation model

(2) $$\begin{array}{r} \underset{X}{\min}||Y-YX|{|}_{F}^{2}+\beta ||X|{|}_{1}. \end{array}$$

where ||.||_*F*_ is the Frobenius norm, *Y* denotes the feature set of all samples (each row denotes a feature and each column represents the feature vector of a sample), *X* is the sparse self-representation coefficient matrix of the columns of *Y* (each column *X*_·*j*_ of *X* denotes the representation coefficient of *j*-th sample *Y*_·*j*_, with all samples in *Y* as dictionary) and β is a tuning parameter to control the trade off between the minimization error and the sparsity. By solving the above model (2), *X* can capture the most similar relationships among the columns of *Y*, based on the information provided in *Y*. In this study, *Y* ∈ {0, 1}^*m* × *n*^ describes the observed associations between lncRNAs and diseases and we would like to predict potential associations between lncRNAs and diseases based on their intra-similarities learned from *Y*. Thus, instead of just finding the representations of the columns of *Y*, we prefer to explore the representations of the rows and columns of *Y* simultaneously, which capture the intra-similarities within lncRNAs and diseases respectively. Based on the idea of sparse self-representation, we introduce a novel two-side sparse self-representation (TSSR) model to handle the task of lncRNA-disease association prediction. In particular, we formulate the framework of TSSR into the following optimization problem

(3) $$\begin{array}{r} \begin{array}{c} \underset{U,V}{\min}||Y-UYV|{|}_{F}^{2}+\beta \left(||U|{|}_{1}+||V|{|}_{1}\right),\\ s.t.\;U\geq 0,V\geq 0,\overset{m}{\underset{z=1}{\sum }}U_{iz}=1,\overset{n}{\underset{k=1}{\sum }}V_{kj}=1. \end{array} \end{array}$$

where $U=\left[U_{ii^{\prime }}\right]\in {\mathbb{R}}_{+}^{m\times m}$ and $V=\left[V_{jj^{\prime }}\right]\in {\mathbb{R}}_{+}^{n\times n}$ are two non-negative sparse matrices which represent the row and column representation coefficient matrices of *Y*, respectively, and β is a tuning parameter which controls the sparsity of *U* and *V*. Based on this definition, *U* denotes the coefficient matrix based on the dictionary *YV*, which captures the similarities among lncRNAs. For example, $U_{ii^{\prime }}$ denotes the similarities between the *i*-th and *i*′-th lncRNAs, which correspond to the *i*-th and *i*′-th rows of *Y*. On the other hand, *V* denotes the coefficient matrix based on the dictionary *UY*, which captures the similarities among diseases. For example, $V_{jj^{\prime }}$ denotes the similarities between the *j*-th and *j*′-th diseases, which correspond to the *j*-th and *j*′-th columns of *Y*. With the sparse regularization term, we can control the sparsity of the learned representation matrices *U* and *V*, and find the most similar relationships within lncRNAs and diseases. The constraints $\overset{m}{\underset{z=1}{\sum }}U_{iz}=1$ and $\overset{n}{\underset{k=1}{\sum }}V_{kj}=1$ are used to guarantee the probability properties of *U*_*i*·_ and *V*_·*j*_, respectively.

In the above objective function (3), the representation matrices are learned from the original data matrix *Y*, which means that they will be sensitive to the input data *Y*. If the input data only includes a small number of known associations, it may be hard to learn a comprehensive representation matrix. With the development of high-throughput experimental techniques and the accumulation of clinical information, we could also collect some functional annotations and phenotype information for lncRNAs and diseases respectively. Based on these prior information, we can infer the intra-associations among diseases and lncRNAs. To utilize these pairwise associations inferred from other databases to promote the estimation of two representation coefficient matrices *U* and *V*, two regularization terms are added to Equation (3). Moreover, we introduce a weight matrix *W* in a similar way to Zheng et al. (2013) to prevent unknown instances (for which association information is not available) from contributing to the determination of the row and column representations of *Y* (i.e., *U* and *V*). The final objective function of our TSSR model is as follows.

(4) $$\begin{array}{l} \begin{array}{c} \begin{array}{c} \underset{U,V}{\min}||W⊙\left(Y-UYV\right)|{|}_{F}^{2}+\beta \left(||U|{|}_{1}+||V|{|}_{1}\right)\\ \;+\lambda_{d}||S_{d}-U|{|}_{F}^{2}+\lambda_{t}||S_{t}-V|{|}_{F}^{2},\\ \;s.t.\;U\geq 0,V\geq 0,\overset{m}{\underset{z=1}{\sum }}U_{iz}=1,\overset{n}{\underset{k=1}{\sum }}V_{kj}=1. \end{array} \end{array} \end{array}$$

where λ_*d*_ and λ_*t*_ are two tuning parameters controlling the influences of prior intra-associations among lncRNAs and diseases, $S_{d}\in {\mathbb{R}}^{m\times m}$ and $S_{t}\in {\mathbb{R}}^{n\times n}$ denote the affinity matrices of lncRNA and disease respectively, where ${\left(S_{d}\right)}_{ii^{\prime }}$ describes the association between lncRNAs *d*_*i*_ and $d_{i^{\prime }}$, and ${\left(S_{t}\right)}_{jj^{\prime }}$ describes the associations between diseases *t*_*j*_ and $t_{j^{\prime }}$. ⊙ denotes the element-wise product or Hadamard product of two matrices and *W* ∈ ℝ^*m* × *n*^ is a weight matrix where *W*_*ij*_ = 0 for unknown entries in *Y* and *W*_*ij*_ = 1 for known entries in *Y*. Consequently, unknown entries in *Y* do not contribute to the minimization of the first term of Equation (4).

### 2.3. Optimization Algorithm

Here, to handle the constraints in (4), we employ a relaxed Majorization-Minimization algorithm (Yang and Oja, 2011, 2012) to obtain the solution of objective function (4). For more details about this optimization method, please refer to Yang and Oja (2012). In particular, we denote ▽_*U*_ as the gradient of our objective function with respect to *U*.

(5) $$\begin{array}{r} \begin{array}{c} {▽}_{U}=-2\left[W⊙\left(Y-UYV\right)\right]V^{\text{T}}Y^{\text{T}}-2\lambda_{d}\left(S_{d}-U\right)+\beta . \end{array} \end{array}$$

Let ${▽}_{U}^{+}=2W⊙\left(UYV\right)V^{\text{T}}Y^{\text{T}}+2\lambda_{d}U+\beta$ and ${▽}_{U}^{-}=2\left(W⊙Y\right)V^{\text{T}}Y^{\text{T}}+2\lambda_{d}S_{d}$ denote the positive and negative parts of ▽_*U*_, respectively. Thus, we have ${▽}_{U}={▽}_{U}^{+}-{▽}_{U}^{-}$.

Due to the constraint $\sum_{z=1}^{m}U_{iz}=1$ and *U*_*iz*_ ≥ 0, we obtain the following updating rule for *U*_*iz*_:

(6) $$\begin{array}{r} U_{iz}^{\text{new}}=U_{iz}\cdot \frac{a_{i}^{U}{\left({▽}_{U}^{-}\right)}_{iz}+1}{a_{i}^{U}{\left({▽}_{U}^{+}\right)}_{iz}+b_{i}^{U}}. \end{array}$$

where $a_{i}^{U}$ and $b_{i}^{U}$ can be obtained by Equations (7) and (8), respectively.

(7) $$\begin{array}{r} a_{i}^{U}=\underset{z}{\sum }\frac{U_{iz}}{{\left({▽}_{U}^{+}\right)}_{iz}}, \end{array}$$

(8) $$\begin{array}{r} b_{i}^{U}=\underset{z}{\sum }U_{iz}\frac{{\left({▽}_{U}^{-}\right)}_{iz}}{{\left({▽}_{U}^{+}\right)}_{iz}}. \end{array}$$

Similarly, we denote ▽_*V*_ as the gradient of our objective function with respect to *V*.

(9) $$\begin{array}{r} \begin{array}{c} {▽}_{V}=-2\left(Y^{\text{T}}U^{\text{T}}\right)\left[W⊙\left(Y-UYV\right)\right]-2\lambda_{t}\left(S_{t}-V\right)+\beta . \end{array} \end{array}$$

Let ${▽}_{V}^{+}=2Y^{\text{T}}U^{\text{T}}\left[W⊙\left(UYV\right)\right]+2\lambda_{t}V+\beta$ and ${▽}_{V}^{-}=2Y^{\text{T}}U^{\text{T}}\left(W⊙Y\right)+2\lambda_{t}S_{t}$ denote the positive and negative parts of ▽_*V*_, respectively, we have ${▽}_{V}={▽}_{V}^{+}-{▽}_{V}^{-}$.

Similarly, the updating rule for *V*_*kj*_ is as follows:

(10) $$\begin{array}{r} V_{kj}^{\text{new}}=V_{kj}\cdot \frac{a_{j}^{V}{\left({▽}_{V}^{-}\right)}_{kj}+1}{a_{j}^{V}{\left({▽}_{V}^{+}\right)}_{kj}+b_{j}^{V}}. \end{array}$$

where $a_{j}^{V}=\underset{k}{\sum }\frac{V_{kj}}{{\left({▽}_{V}^{+}\right)}_{kj}}$ and $b_{j}^{V}=\underset{k}{\sum }V_{kj}\frac{{\left({▽}_{V}^{-}\right)}_{kj}}{{\left({▽}_{V}^{+}\right)}_{kj}}$.

The details of the optimization algorithm to the proposed TSSR model are described in Algorithm 1. *U* and *V* can be updated by Equations (6) and (10), respectively. In this study, we stop the iteration when the changes of *U* and *V* are less than 1e-6, measured by *L*_1_ norm. Finally, the predicted label matrix Ŷ can be returned by Ŷ = *UYV* when algorithm arrives at the convergence conditions.

**Algorithm 1:** Algorithm for the TSSR model

**Inputs:** Partial label matrix *Y*, lncRNA affinity matrix *S*_*d*_, disease affinity matrix *S*_*t*_, tuning parameter λ_*d*_, λ_*t*_, β, weight matrix *W*. **Output:** Predicted label matrix Ŷ. **Main algorithm:** Initialize *U* and *V*; **While** not converged **do** Update *U* according to Equation (6) $U_{iz}^{\text{new}}=U_{iz}\cdot \frac{a_{i}^{U}{\left({▽}_{U}^{-}\right)}_{iz}+1}{a_{i}^{U}{\left({▽}_{U}^{+}\right)}_{iz}+b_{i}^{U}}$; Update *V* according to Equation (10) $V_{kj}^{\text{new}}=V_{kj}\cdot \frac{a_{j}^{V}{\left({▽}_{V}^{-}\right)}_{kj}+1}{a_{j}^{V}{\left({▽}_{V}^{+}\right)}_{kj}+b_{j}^{V}}$; Check the convergence conditions. **End while** **Return** Ŷ = *UYV*.

## 3. Results

In this section, we demonstrate the performance of various algorithms on three real datasets. Furthermore, case studies of three cancer diseases (i.e., Melanoma, Glioblastoma, and Glioma) are performed to validate the effectiveness of our TSSR model. The materials, experimental settings, and parameter settings are described as follows.

### 3.1. Materials

#### 3.1.1. LncRNA-Disease Associations

We collect three datasets to evaluate the performance of various prediction algorithms. The first dataset is downloaded from the supplementary data of a article (Lu et al., 2018), which contains 621 experimentally confirmed lncRNA-disease associations between 226 diseases and 285 lncRNAs from the LncRNADisease database^1^ established in 2015. The second dataset involving 260 high-quality associations between 95 lncRNAs and 81 human disease is obtained from the supplementary files of the published article (Chen et al., 2015), which retrieved data from MNDR database^2^ (Wang et al., 2013) in March 2015. The third dataset is downloaded from the Lnc2Cancer database ^3^ in 2015. By getting rid of the duplicate lncRNA-disease associations for the same lncRNA-disease pair, we obtain 677 distinct associations, including 54 human cancers and 436 lncRNAs. The statistics of the three datasets are illustrated in Table 1.

**Table 1:** The statistics of three datasets.

**Datasets**	**No.of lncRNA**	**No.of disease**	**No.of associations**	**Density**
LncRNA Disease	285	226	621	0.01
MNDR	95	81	260	0.03
Lnc2Cancer	436	54	677	0.03

#### 3.1.2. Disease Similarities

As previous studies have discovered that diseases with similar phenotypes are usually related with similar dysfunctions of lncRNAs (Chen et al., 2015), incorporating the similarities among diseases estimated from other database may help to infer the potential associations between diseases and lncRNAs based on known lncRNA-disease associations. Similar to previous studies (Wang et al., 2010; Chen et al., 2015), we construct the similarity matrix *S*_*t*_ of diseases by integrating the disease semantic similarity matrix inferred from the structure of directed acyclic graph that describes the relationships among diseases (Wang et al., 2010; Chen et al., 2015) and disease Gaussian interaction profile kernel similarity matrix inferred from known associations between diseases and lncRNAs (Chen and Yan, 2013; Chen et al., 2015). In particular, we obtain the similarity matrix *S*_*t*_ by averaging the disease similarity matrix and disease Gaussian interaction profile kernel similarity matrix (van Laarhoven et al., 2011; Chen and Yan, 2013; Chen et al., 2015, 2016a).

#### 3.1.3. LncRNA Similarities

Since lncRNAs with similar functions tend to exhibit similar associations with diseases, calculating the similarities among lncRNAs will promotes the identification of potential associations between diseases and lncRNAs. In this study, we calculate the similarity matrix *S*_*d*_ of lncRNAs by integrating the functional similarity matrix calculated by the model of LNCSIM (Chen et al., 2015) and the lncRNA Gaussian interaction profile kernel similarity matrix estimated from known associations between lncRNAs and diseases (Chen and Yan, 2013). Similar to the disease similarity matrix *S*_*t*_, we obtain the lncRNA similarity matrix *S*_*d*_ by averaging the lncRNA functional similarity matrix and Gaussian interaction profile kernel similarity matrix (van Laarhoven et al., 2011; Chen and Yan, 2013; Chen et al., 2015; Chen et al., 2016a).

### 3.2. Experimental Settings

To illustrate the effectiveness of our proposed TSSR model, we compare our method with other six state-of-the-art association prediction methods, namely NetlapRLS (Xia et al., 2010), BLM-NII (Mei et al., 2012), CMF (Zheng et al., 2013), PBMDA (You et al., 2017a), PRMDA (You et al., 2017b), and SIMCLDA (Lu et al., 2018). All these methods are designed for predicting the inter-associations between different types of biological entities and all of them can make use of the prior intra-associations among biological entities to improve their performance. Thus, all these algorithms are well suited for undertaking the task of lncRNA-disease association prediction. Moreover, our experiment results show that they are effective in inferring the associations between diseases and lncRNAs. Specifically, 15 repetitions of 10-fold cross validation (CV) are conducted for each model, with receiver operating characteristic (ROC) curve as the main metric to evaluate the performance. By stacking the columns of matrix *Y*, we obtained the vector, a *mn* × 1 vector, denoted as vec(*Y*). In each repetition of 10-fold CV, we divide vec(*Y*) into ten disjoint folds randomly. Nine folds are treated as the training set while the remaining one fold is left out as the testing set. The AUC (Area Under Curve) score is calculated for each 10-fold CV repetition, and the final AUC score for each model are obtained by averaging over 15 such repetitions.

### 3.3. Parameter Settings

As each model has some hyperparameters that need to be predefined, we perform cross validation on the training set to determine the values of these hyperparameters. In particular, the parameter settings for various models are described as follows. For NetLapRLS (Xia et al., 2010), the hyperparameters satisfy $\frac{\gamma_{d2}}{\gamma_{d1}}$=$\frac{\gamma_{p2}}{\gamma_{p1}}$, β_*d*_ = β_*p*_ with their values chosen from {10^−6^, 10^−5^, …, 10^2^}. For BLM-NII (Mei et al., 2012), the value of the linear combination weight α is chosen from {0, 0.1, 0.2, …, 1.0}. The max function is utilized to combine the interaction scores inferred from the disease and lncRNA sides. For the matrix factorization based methods, the dimensionality of the latent space *K* is selected from {50, 100} (Zheng et al., 2013). For CMF (Zheng et al., 2013), the regularization coefficient λ_1_ is chosen from {2^−2^, …, 2^1^} (Zheng et al., 2013), while the values of λ_*d*_ and λ_*t*_ are chosen from {2^−3^, 2^−2^, …, 2^5^}. For PBMDA (You et al., 2017a), the maximum path length *L* is set to 3 and the weight threshold *T* is selected from {0.2, 0.3, …, 0.8} with the step size set to 0.1, while the decay factor α is set to 2.26. For SIMCLDA (Lu et al., 2018), we set the values of α_*l*_ and α_*d*_ from 0.1 to 1 with stepsize 0.1 and select the regularization parameter from {10^−3^, 10^−2^, …, 10^3^}. For TSSR, we choose the three parameters β and λ_*d*_ = λ_*t*_ from {2^−10^, 2^−9^, …, 2^9^, 2^10^}. Note that the most suitable hyper-parameters of a machine learning model on different datasets are usually different. Therefore, in this work, we adopt grid search (Bergstra and Bengio, 2012) to select the optimal hyperparameters for each model on each dataset.

### 3.4. Comparison With State-of-the-Art Methods

**Figure 1:**
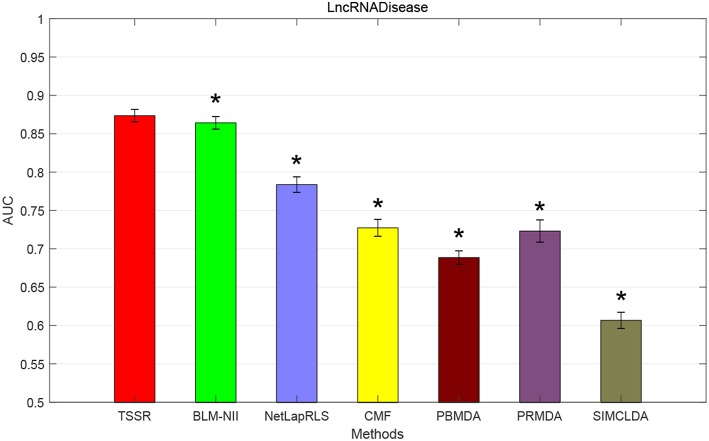
AUC scores of various algorithms in LncRNADisease dataset (* indicates TSSR significantly outperforms the competitor with *p* < 0.05 using *t*-test, error bars denote 95% confidence intervals).

**Figure 2:**
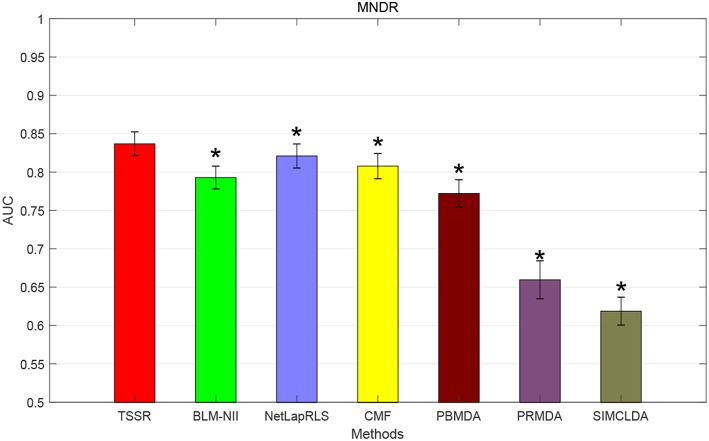
AUC scores of various algorithms in MNDR dataset (* indicates TSSR significantly outperforms the competitor with *p* < 0.05 using *t*-test, error bars denote 95% confidence intervals).

**Figure 3:**
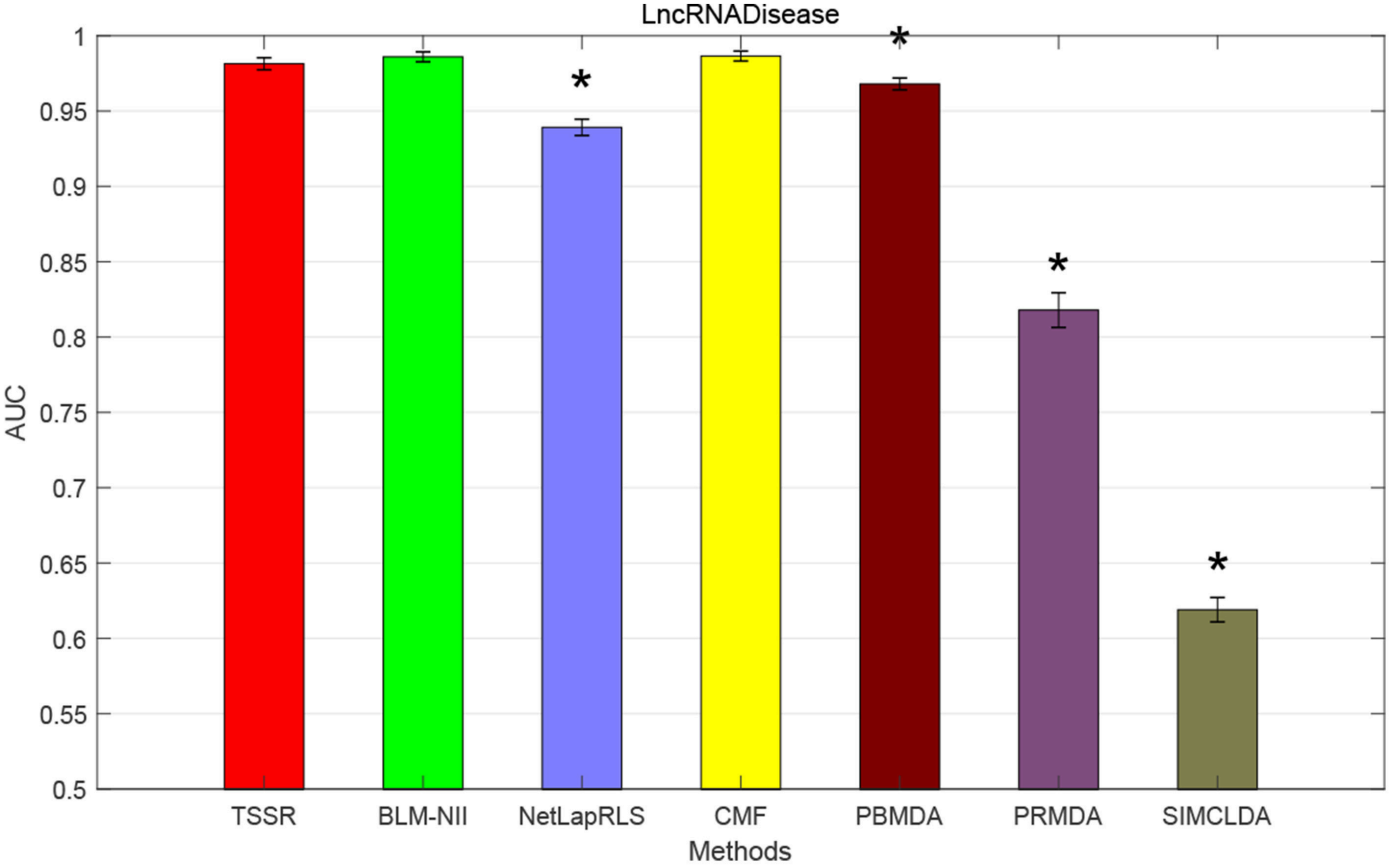
AUC scores of various algorithms in Lnc2Cancer dataset (* indicates TSSR significantly outperforms the competitor with *p* < 0.05 using *t*-test, error bars denote 95% confidence intervals).

**Figure 4:**
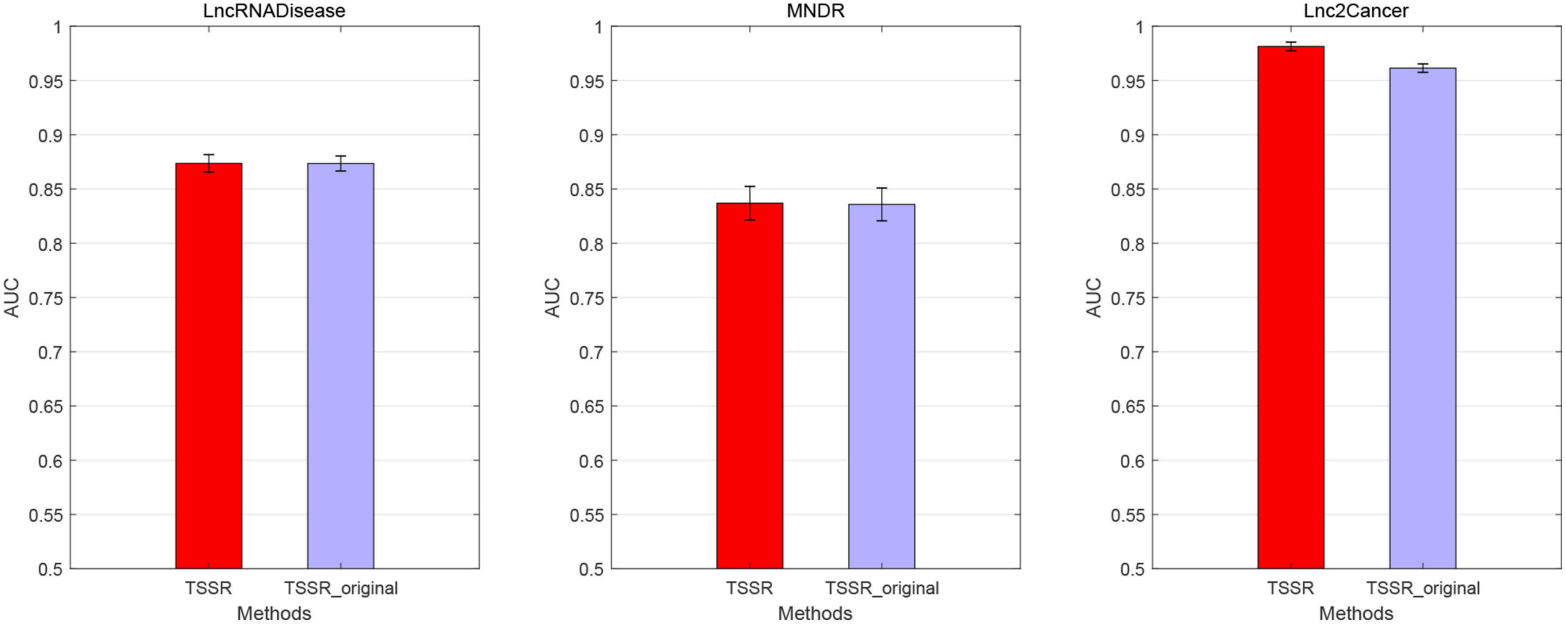
Performance of TSSR with and without external information (denoted by TSSR and TSSR_original, respectively) on LncRNADisease, MNDR, and Lnc2Cancer datasets, measured by AUC (error bars denote 95% confidence intervals).

We conduct the experiments with 10-fold CV to shed light on the performance of TSSR in predicting potential lncRNA-disease associations, compared with other six state-of-the-art methods. Here, the AUC score is used to evaluate the predictive performance of various methods. The experiment results measured by AUC are shown in Figures 1–3. As shown in Figure 1, on LncRNADisease dataset, TSSR obtains an AUC score of 0.8736, which is higher than other methods (BLM-NII 0.8641, NetLapRLS 0.7837, CMF 0.7273, PBMDA 0.6885, PRMDA 0.7231, SIMCLDA 0.6067), indicating the superiority of our TSSR in predicting lncRNA-disease associations. We can find from Figure 2 that on MNDR dataset, TSSR achieves the best AUC score (TSSR 0.8369, BLM-NII 0.7929, NetLapRLS 0.8210, CMF 0.8078, PBMDA 0.7722, PRMDA 0.6596, SIMCLDA 0.6187). On Lnc2Cancer dataset (the results are shown in Figure 3), TSSR still has competitive performance with other six methods with respect to AUC score (TSSR 0.9814, BLM-NII 0.9859, NetLapRLS 0.9392, CMF 0.9864, PBMDA 0.9680, PRMDA 0.8179, SIMCLDA 0.6190). Note that on Lnc2Cancer, our TSSR achieves similar performance with BLM-NII and CMF. This may due to the parameter setting of TSSR. In this study, the values of the hyperparameters λ_*d*_ and λ_*t*_ (which control the influences of prior intra-similarities among lncRNAs and diseases) in our TSSR are set to same for simplicity, which is reasonable when the two data sets are balanced. However, the number of lncRNAs and diseases in Lnc2Cancer dataset are imbalanced. Thus, forcing λ_*d*_ and λ_*t*_ to be equal may limit the performance of TSSR. If the values of λ_*d*_ and λ_*t*_ are tuned separately, TSSR could achieve better performance. Moreover, to evaluate the effect of external information on the performance of TSSR, we remove the regularization terms related to the external information (i.e., setting λ_*d*_ = λ_*t*_ = 0) and show the results in Figure 4. As shown in this figure, the performance of TSSR and TSSR without external information (denoted by TSSR_original) is comparable (on LncRNADisease, TSSR 0.8736, TSSR_original 0.8735; on MNDR, TSSR 0.8369, TSSR_original 0.8367; on Lnc2Cancer, TSSR 0.9814, TSSR_original 0.9614), which means the improved performance of TSSR is mainly due to the self-representation learning. Thus, our TSSR does not depend heavily on the external information. All these results demonstrate the effectiveness of the proposed TSSR in predicting potential lncRNA-disease associations.

### 3.5. Effects of Parameters

The proposed TSSR involves three parameters, λ_*d*_, λ_*t*_, and β, where λ_*d*_ and λ_*t*_ control the influences of prior intra-associations among lncRNAs and diseases and β controls the sparsity of *U* and *V*. We will study how these parameters affect the performance of TSSR.

**Figure 5:**
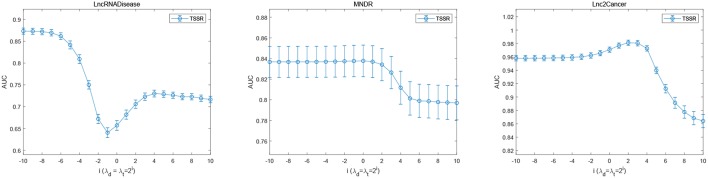
Performance of TSSR on LncRNADisease, MNDR, and Lnc2Cancer datasets, measured by AUC with different values of λ_*d*_ and λ_*t*_ (error bars denote 95% confidence intervals).

Figure 5 shows the prediction performance of TSSR on LncRNADisease dataset, MNDR dataset and Lnc2Cancer dataset, measured by AUC with respect to different values of λ_*d*_ and λ_*t*_. As shown in Figure 5, the optimal value of λ_*d*_ = λ_*t*_ for these three datasets is 2^−10^, 2^0^, and 2^2^, respectively, while β is set to 2^1^, 2^8^, and 2^8^, respectively. We find that TSSR usually performs well when the values of λ_*d*_ and λ_*t*_ are relatively small, which means the additional use of external information is not always helpful for performance improvement. On the contrary, if the external information contains noise, the performance of TSSR may decrease if we overemphasizing the effect of external information. These results demonstrate that our TSSR can effectively learn the representation matrices from known lncRNA-disease associations, and flexibly utilize external information to promote the prediction of potential lncRNA-disease associations.

**Figure 6:**
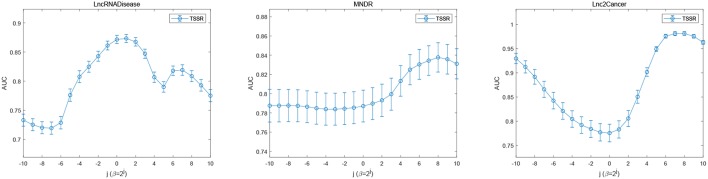
Performance of TSSR on LncRNADisease, MNDR, and Lnc2Cancer datasets, measured by AUC with different values of β (error bars denote 95% confidence intervals).

In addition, we also study the impact of sparsity control parameter β. Figure 6 illustrates the AUC scores obtained by TSSR in terms of different values of β. As shown in Figure 6, on these three datasets, TSSR achieves the best AUC score when the value of β is 2^1^, 2^8^, and 2^8^, respectively, while λ_*d*_ = λ_*t*_ is set to 2^−10^, 2^0^, and 2^2^, respectively. We can also find from this figure that larger values of β can generally achieve better performance, which indicates the importance of controlling the sparsity of the representation matrices *U* and *V*.

### 3.6. Case Studies

To further validate the performance of our algorithm, based on the LncRNADisease dataset, we apply our TSSR model to identify the most possible lncRNAs that associated with three cancers (i.e., Melanoma, Glioma, and Glioblastoma). Here, all the known associations in the LncRNADisease dataset are used to train the model. Then we select the top 20 associated lncRNAs which get the highest predicted ranks for each cancer and verify these predictions based on MNDR and Lnc2Cancer databases. Moreover, the relevant literatures that support the prediction results are listed to indicate whether the predicted lncRNA-disease associations have been experimentally validated. Specially, MNDR database contains both experimental and prediction evidence (Ning et al., 2016; Ping et al., 2018). The results for the three cancers are shown in Tables 2–**4**, respectively. Note that we only show the predictions that are not included in the training set.

**Table 2:** The identified novel lncRNAs that have been verified to be associated with Melanoma.

**Rank**	**lncRNA**	**Evidence(Database)**	**Evidence(PMID)**	**Expression pattern**
1	CCAT2	MNDR	Prediction evidence	
2	TUSC7	MNDR	Prediction evidence	
9	GHET1	MNDR	Prediction evidence	
12	MEG3	MNDR/lnc2Cancer	29781534,29808164	Up-regulated, differential expression
13	HOTAIR	MNDR/lnc2Cancer	28067428,23862139	up-regulated
14	SOX2-OT	MNDR	Prediction evidence	
15	MALAT1	MNDR/lnc2Cancer	27725873, 27564100,27966454,24892958,19625619	Up-regulated,differential expression
17	SNHG5	MNDR/lnc2Cancer	26440365	Up-regulated
18	BCAR4	MNDR	Prediction evidence	
19	CCAT1	lnc2Cancer	28409554	Up-regulated

*Prediction evidence denotes the prediction associations in MNDR database*.

Melanoma is a deadly malignancy which develops from the pigment-containing cells with increasing incidence than that of any other types of cancer (Aladowicz et al., 2013). People with low level of skin pigment exposure in excess ultraviolet light (UV) have a high risk to be infected a melanoma (Kanavy and Gerstenblith, 2011). It has been estimated that by 2030, melanoma could overtake colorectal cancer as the fifth most common cancer (Rahib et al., 2014). Therefore, we apply our TSSR model to predict the potential melanoma-associated lncRNAs. According to the results shown in Table 2 (the complete list of the top 20 identified lncRNAs is shown in Supplementary Material), 10 out of the top 20 identified lncRNAs have been verified. For example, Luan et al. (2016) discovered that MALAT1 could promote the cell proliferation, invasion and migration of melanoma. Li et al. observed that MEG3 was obviously decreased in melanoma cells (Li et al., 2018). They also found melanoma cell apoptosis was induced by up-regulation of MEG3, and consequently come to a conclusion that overexpression of MEG3 has a significant repression impact in melanoma cell migration and invasion ability.

Glioma is one of the most common primary malignant tumors originating in the brain, which comprises approximately 30% of all brain tumors (Goodenberger and Jenkins, 2012; Boele et al., 2015). Glioma can be graded from I to IV by World Health Organization (WHO) grading system according to their grade (Louis et al., 2016a,b). The exact causes of glioma are still unclear at the present (Kwiatkowska and Symons, 2013; Li et al., 2015). Studies have revealed the roles of lncRNAs in the development of human disease, including glioma (Zhou et al., 2018). Here, we utilize the TSSR to identify the potential lncRNAs that are more likely to related to glioma. Based on the experiment results, 9 out of the top 20 identified lncRNAs have been validated in the MNDR and Lnc2Cancer databases, and other relevant literatures. The results are shown in Table 3 (the complete list of the top 20 identified lncRNAs is shown in Supplementary Material). For example, Ma et al. discovered that compared with paired normal tissues, the expression level of lncRNA MALAT1 was increased in glioma tissues, which means MALAT1 can be treated as a convictive marker for the prognosis of glioma patients (Ma et al., 2015). Zou et al. revealed that glioma patients with high PVT1 expression had low survival rate (Zou et al., 2017). Moreover, patients who received chemotherapy and radiotherapy could improve their survival by down-regulating PVT1. They also indicated that PVT1 could be served as potential target for the treatment of diffuse gliomas.

**Table 3:** The identified novel lncRNAs that have been verified to be associated with Glioma.

**Rank**	**lncRNA**	**Evidence(Database)**	**Evidence(PMID)**	**Expression pattern**
2	HOTAIR	MNDR/lnc2Cancer	29323737,28083786 ,29218099, 27277755,24203894	Up-regulated, down-regulated
3	MALAT1	MNDR/lnc2Cancer	28551849,27134488,26649728,25613066,26619802	Up-regulated, down-regulated
4	GAS5	MNDR/lnc2Cancer	26370254,28666797	Up-regulated, down-regulated
7	PVT1	lnc2Cancer	28351322,29108264,29620147,29501773,29046366	Up-regulated, differential expression
11	SPRY4-IT1	MNDR/lnc2Cancer	29467908,27460732,26464658	Up-regulated
12	GHET1	MNDR	Prediction evidence	
15	IGF2-AS	MNDR	Prediction evidence	
18	LincRNA-p21	lnc2Cancer	28689810	Down-regulated
19	SNHG4	MNDR	Prediction evidence	

*Prediction evidence denotes the prediction associations in MNDR database*.

Glioblastoma, also known as glioblastoma multiform (GBM) (grade IV of Glioma), is the most common and aggressive form of primary brain tumors and kills nearly every patient in a median time of 15 months (Bleeker et al., 2012; Jovčevska et al., 2013). More importantly, there is still no clear way to prevent the disease (Gallego, 2015). Therefore, it is urgent to predict the potential glioblastoma-associated lncRNAs. In this study, we use our TSSR to undertake this task. As shown in Table 4, 8 out of the 20 lncRNAs have been verified in the MNDR and Lnc2Cancer databases, and other relevant literatures (the complete list of the top 20 identified lncRNAs is shown in Supplementary Material). For example, Zhou et al. described that HOTAIR has a significant increased expression in multiple human cancers including GBM and they found HOTAIR is necessary for GBM formation *in vivo* (Zhou X. et al., 2015). Thus, HOTAIR could be a potential therapeutic target in glioblastoma. Liu et al. found that NBAT1 has lower expressions in glioblastoma tissues compared with those in normal brain tissues and they also observed that up-regulated NBAT1 inhibits proliferation of T98 and U87 cells via regulating Akt, suggesting that NBAT1 may be related to prognosis of glioblastoma (Liu et al., 2018).

**Table 4:** The identified novel lncRNAs that have been verified to be associated with Glioblastoma.

**Rank**	**lncRNA**	**Evidence(Database)**	**Evidence(PMID)**	**Expression pattern**
1	MEG3	MNDR/lnc2Cancer	27306825,28187000,22234798,25378224,26111795	Up-regulated
2	HOTAIR	MNDR/lnc2Cancer	27306825,25428914,25823657,26111795,26943771	Up-regulated
6	BCYRN1	MNDR	25561975	Differentially expressed
8	GAS5	MNDR/lnc2Cancer	27784795,23726844	Up-regulated, differentially expressed
10	NEAT1	lnc2Cancer	23046790	Up-regulated
11	HIF1A-AS2	MNDR/lnc2Cancer	27264189	Up-regulated
15	NBAT1	lnc2Cancer	29771423	Up-regulated
17	NDM29	MNDR	25561975	Differentially expressed

*Prediction evidence denotes the prediction associations in MNDR database*.

Based on the above case studies, we find that our TSSR is effective in identifying novel associations between lncRNAs and diseases based on known lncRNA-disease associations and intra-associations among lncRNAs and diseases.

## 4. Conclusion

Increasing evidences indicate the role of lncRNAs in biological processes, which motivates the development of computational models to identify the potential associations between lncRNAs and diseases. Predicting the potential associations between lncRNAs and diseases based on known lncRNA-disease associations is equivalent to a recommendation problem with implicit feedback, where the task is to predict whether the unknown pairs in *Y* are potential associations or not. In this paper, we present a novel model, named two-side sparse self-representation (TSSR), to predict the scores of unknown pairs in *Y*. Based on these predicted scores, we could identify potential associations between lncRNAs and diseases. Unlike previous matrix factorization techniques that project lncRNAs and diseases into a shared latent space and predict lncRNA-disease associations based on the inner product of their latent vectors (where the dimension of latent space is previously unknown and hard to determine), our model directly learn the intra-similarities among lncRNAs and diseases from the observed associations in *Y*, and utilize the learned representation matrices to reconstruct *Y* by regarding original *Y* as a dictionary. As shown in Equation (4), our TSSR does not need to make many assumptions of the model in advance. Moreover, by forcing the representation matrices to be sparse, our TSSR could learn the most similar relationships among lncRNAs and diseases based on the observed associations in *Y*. Thus, our TSSR has data-adaptiveness and avoids the determination of some sensitive parameters such as the dimension of latent space and number of nearest neighbors. Unlike random walk-based or data integration-based methods that rely heavily on the similarity networks inferred from external information with predefined metrics, our model could adaptively learn the self-representations of lncRNAs and diseases according to their performance in reconstructing observed associations in *Y*. Moreover, in case the input data *Y* only includes a small number of known associations, our model could draw support from the intra-associations among lncRNAs and diseases derived from external information to enhance the learning of representation matrices. Therefore, our model could effectively predict potential lncRNA-disease associations by leveraging the information provided by known lncRNA-disease associations and external information of lncRNAs and diseases. Experiment results on three real data sets show that our TSSR could achieve better performance than other six state-of-the-art methods. The effectiveness of TSSR in predicting potential lncRNA-disease associations is also evaluated based on three case studies. As a link prediction algorithm, our TSSR model is flexible and could be used to handle other link prediction tasks in bipartite networks.

Furthermore, since external information of lncRNAs and diseases are utilized to enhance the performance of various methods, we also perform sensitivity analysis to assess the influences of noise information on the performances of various methods. In particular, we generate the similarity matrices *S*_*d*_ and *S*_*t*_ randomly (i.e., the elements in *S*_*d*_ and *S*_*t*_ are generated randomly) and test the performances of various methods. The detailed experiment results are shown in Tables S4–S6. As shown in these tables, although the performance of TSSR is affected by the noise information, it could still achieve the best performance, which means our TSSR could be used to undertake the lncRNA-disease prediction task even when the collected external information of lncRNAs and diseases contains a lot of noise.

With the development of high-throughput experimental techniques, an increasing number of data for lncRNAs and diseases are becoming available. We can calculate the similarities among lncRNAs (or diseases) based on different views of data and different metrics. How to efficiently seek the optimal combination of these similarities is an interesting future work. We will try to extend our model to handle this problem.

## Author Contributions

LO-Y and JH conceived and designed the study, performed the statistical analysis, and drafted the manuscript. ZZ conceived of the study, and participated in its design and coordination and helped to draft the manuscript. X-FZ and Y-RL participated in the design of the study, performed the statistical analysis, and helped to revise the manuscript. YS and SH participated in the design of the study and helped to revise the manuscript. All authors read and approved the final manuscript.

### Conflict of Interest Statement

The authors declare that the research was conducted in the absence of any commercial or financial relationships that could be construed as a potential conflict of interest.

## Footnotes

^1^http://www.cuilab.cn/lncrnadisease

^2^http://www.rna-society.org/mndr/

^3^http://www.bio-bigdata.com/lnc2cancer/
