# Supplementary material for: LncRNA-Disease Association Prediction Using Two-Side Sparse Self-Representation
Source: Front Genet. 2019 May 28;10:476. doi: 10.3389/fgene.2019.00476 (PMC6546878; doi:10.3389/fgene.2019.00476)
Supplement: Supplementary file 1 [file Data_Sheet_1.PDF]

Supplementary Material: LncRNA-Disease Association Prediction using Two-Side Sparse Self-Representation

1 SUPPLEMENTARY DATA

1.1 Sensitivity Analysis

Since external information of lncRNAs and diseases are utilized to enhance the performance of various methods, we perform sensitivity analysis to assess the influences of noise information on the performances of various methods. In particular, we generate the similarity matrices S_d and S_t randomly (i.e., the elements in S_d and S_t are generated randomly) and test the performances of various methods. The experiment results are shown in Tables S4, S5 and S6. We can find from these tables that the performances of all methods are degraded when the similarity matrices S_d and S_t are generated randomly. As shown in these tables, although the performance of TSSR is affected by the noise information, it could still achieve the best performance, which means our TSSR could be used to undertake the lncRNA-disease prediction task even when the collected external information of lncRNAs and diseases contains a lot of noise.

2 SUPPLEMENTARY TABLES

Table S1. The identified novel lncRNAs that have been verified to be associated with Melanoma. Prediction evidence denotes the prediction associations in MNDR database.

Rank	lncRNA	Evidence(Database)	Evidence(PMID)
1	CCAT2	MNDR	prediction evidence
2	TUSC7	MNDR	prediction evidence
3	GAPLINC		
4	ESCCAL-1		
5	MINA		
6	PTENpg1		
7	SUMO1P3		
8	ESCCAL-5		
9	GHET1	MNDR	prediction evidence
10	MALAT2		
11	lncRNA-MVIH		
12	MEG3	MNDR/lnc2Cancer	29781534,29808164
13	HOTAIR	MNDR/lnc2Cancer	29156728,28067428,23862139
14	SOX2-OT	MNDR	prediction evidence
15	MALAT1	MNDR/lnc2Cancer	27725873, 27564100,27966454,24892958,19625619
16	ZFAT-AS1		
17	SNHG5	MNDR/lnc2Cancer	26440365
18	BCAR4	MNDR	prediction evidence
19	CCAT1	lnc2Cancer	28409554
20	Loc554202		

Table S2. The identified novel lncRNAs that have been verified to be associated with Glioma. Prediction evidence denotes the prediction associations in MNDR database.

Rank	lncRNA	Evidence(Database)	Evidence(PMID)
1	PTENpg1		
2	HOTAIR	MNDR/lnc2Cancer	29323737,28083786 ,29218099, 27277755,24203894
3	MALAT1	MNDR/lnc2Cancer	28551849,27134488,26649728,25613066,26619802
4	GAS5	MNDR/lnc2Cancer	26370254,28666797
5	MINA		
6	anti-NOS2A		
7	PVT1	lnc2Cancer	28351322,29108264,29620147,29501773,29046366
8	NBAT17		
9	HTTAS		
10	MYCNOS		
11	SPRY4-IT1	MNDR/lnc2Cancer	29467908,27460732,26464658
12	GHET1	MNDR	prediction evidence
13	SUMO1P3		
14	NDM29		
15	IGF2-AS	MNDR	prediction evidence
16	GAPLINC		
17	7SK		
18	LincRNA-p21	lnc2Cancer	28689810
19	SNHG4	MNDR	prediction evidence
20	PINC		

Table S3. The identified novel lncRNAs that have been verified to be associated with Glioblastoma. Prediction evidence denotes the prediction associations in MNDR database.

Rank	lncRNA	Evidence(Database)	Evidence(PMID)
1	MEG3	MNDR/lnc2Cancer	27306825,28187000,22234798,25378224,26111795
2	HOTAIR	MNDR/lnc2Cancer	27306825,25428914,25823657,26111795,26943771
3	CDKN2B-AS1		
4	HULC		
5	LncRNA-LALR1		
6	BCYRN1	MNDR	25561975
7	KCNQ1OT1		
8	GAS5	MNDR/lnc2Cancer	27784795,23726844
9	TUSC8		
10	NEAT1	lnc2Cancer	23046790
11	HIF1A-AS2	MNDR/lnc2Cancer	27264189
12	SRA1		
13	SNHG167		
14	HIF1A-AS1		
15	NBAT1	lnc2Cancer	29771423
16	MYCNOS		
17	NDM29	MNDR	25561975
18	T-UCRs		
19	LincRNA-p21		
20	MINA		

Table S4. The AUC scores of various algorithms in LncRNADisease dataset with S_d and S_t generated from other databases ("Original results") and generated randomly ("Results with noisy information").

Methods	TSSR	BLM-NII	NetLapRLS	CMF	PBMDA	PRMDA	SIMCLDA
Original results	0.8736	0.8641	0.7837	0.7273	0.6885	0.7231	0.6067
Results with noisy information	0.8480	0.7857	0.7601	0.7137	0.7389	0.6129	0.6235

Table S5. The AUC scores of various algorithms in MNDR dataset with S_d and S_t generated from other databases ("Original results") and generated randomly ("Results with noisy information").

Methods	TSSR	BLM-NII	NetLapRLS	CMF	PBMDA	PRMDA	SIMCLDA
Original results	0.8369	0.7929	0.8210	0.8078	0.7722	0.6596	0.6187
Results with noisy information	0.8002	0.6067	0.7825	0.7481	0.7591	0.6503	0.5944

Table S6. The AUC scores of various algorithms in Lnc2Cancer dataset with S_d and S_t generated from other databases ("Original results") and generated randomly ("Results with noisy information").

Methods	TSSR	BLM-NII	NetLapRLS	CMF	PBMDA	PRMDA	SIMCLDA
Original results	0.9814	0.9859	0.9392	0.9864	0.9680	0.8179	0.6190
Results with noisy information	0.9017	0.8978	0.8202	0.7352	0.8538	0.4957	0.5205
